# Bis(pyridazine-κ*N*)bis­(seleno­cyanato-κ*N*)zinc

**DOI:** 10.1107/S1600536811012621

**Published:** 2011-04-13

**Authors:** Thorben Reinert, Jan Boeckmann, Christian Näther

**Affiliations:** a Institut für Anorganische Chemie, Christian-Albrechts-Universität Kiel, Max-Eyth Strasse 2, D-24098 Kiel, Germany

## Abstract

The asymmetric unit of the title compound, [Zn(NCSe)_2_(C_4_H_4_N_2_)_2_], consists of one Zn^II^ cation, located on a twofold rotation axis, one seleno­cyanate anion and one pyridazine ligand in general positions. The Zn^II^ atom is coordinated by two N-atoms of two pyridazine ligands and two terminal *N*-bonded seleno­cyanate anions within a slightly distorted tetra­hedral coordination environment. In the crystal, discrete complex mol­ecules are arranged in layers parallel to the *ac* plane, with Zn^II^⋯Zn^II^ distances of 8.0906 (6) Å along the *a* axis and of 9.0490 (7) or 9.3604 (7) Å along the *c* axis. The complex mol­ecules are further linked *via* weak Se⋯Se inter­actions, with Se⋯Se distances of 3.8235 (9) Å.

## Related literature

For related structures see: Boeckmann *et al.* (2011 ▶); Bhosekar *et al.* (2010 ▶); Wriedt & Näther (2010 ▶); Zhu *et al.* (2008 ▶).
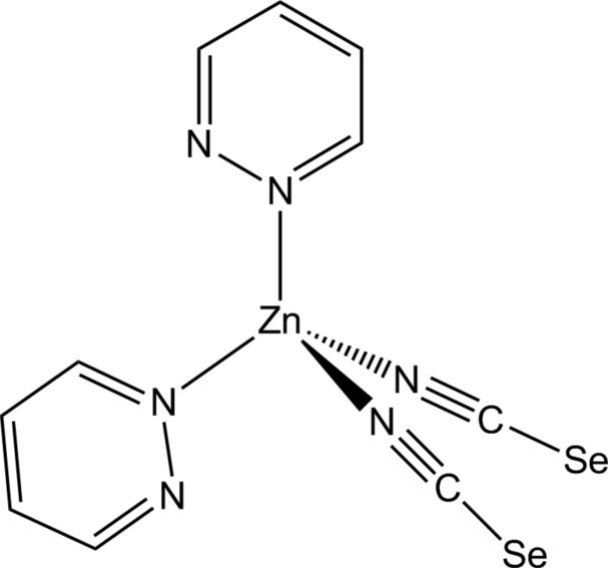


## Experimental

### 

#### Crystal data

[Zn(NCSe)_2_(C_4_H_4_N_2_)_2_]
*M* *_r_* = 435.51
Monoclinic, 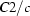

*a* = 15.1521 (10) Å
*b* = 5.6783 (4) Å
*c* = 17.4855 (13) Å
β = 94.981 (6)°
*V* = 1498.74 (18) Å^3^
*Z* = 4
Mo *K*α radiation
μ = 6.49 mm^−1^
*T* = 293 K
0.09 × 0.06 × 0.04 mm

#### Data collection

Stoe IPDS-2 diffractometer
Absorption correction: numerical (*X-SHAPE* and *X-RED32*; Stoe & Cie, 2008 ▶) *T*_min_ = 0.373, *T*_max_ = 0.664
9054 measured reflections
1634 independent reflections
1287 reflections with *I* > 2σ(*I*)
*R*_int_ = 0.028

#### Refinement

*R*[*F* ^2^ > 2σ(*F* ^2^)] = 0.046
*wR*(*F* ^2^) = 0.097
*S* = 1.13
1634 reflections
87 parameters
H-atom parameters constrained
Δρ_max_ = 0.49 e Å^−3^
Δρ_min_ = −0.39 e Å^−3^

### 

Data collection: *X-AREA* (Stoe & Cie, 2008 ▶); cell refinement: *X-AREA*; data reduction: *X-AREA*; program(s) used to solve structure: *SHELXS97* (Sheldrick, 2008 ▶); program(s) used to refine structure: *SHELXL97* (Sheldrick, 2008 ▶); molecular graphics: *XP* in *SHELXTL* (Sheldrick, 2008 ▶) and *DIAMOND* (Brandenburg, 2011 ▶); software used to prepare material for publication: *SHELXL97*.

## Supplementary Material

Crystal structure: contains datablocks I, global. DOI: 10.1107/S1600536811012621/bt5509sup1.cif

Structure factors: contains datablocks I. DOI: 10.1107/S1600536811012621/bt5509Isup2.hkl

Additional supplementary materials:  crystallographic information; 3D view; checkCIF report

## Figures and Tables

**Table:** 

Zn1—N1	1.925 (4)
Zn1—N11	2.022 (3)

**Table:** 

N1—Zn1—N1^i^	117.5 (3)
N1—Zn1—N11^i^	111.40 (17)
N1—Zn1—N11	106.96 (16)
N11^i^—Zn1—N11	101.48 (18)

Symmetry code: (i) 
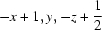
.

**Fig. 1.**
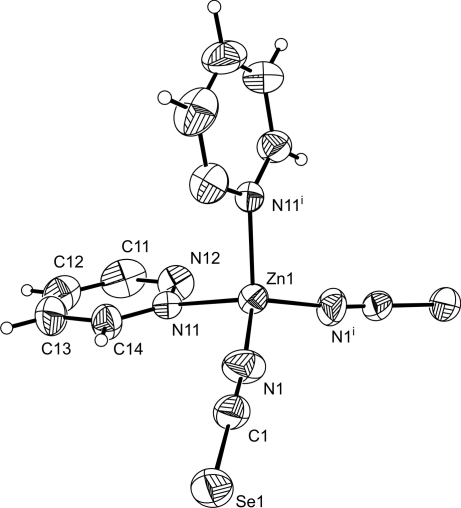
: Crystal structure of the title compund with labelling and displacement ellipsoids drawn at the 30% probability level. Symmetry codes: i = -x + 1, y, -z + 1/2.

**Fig. 2.**
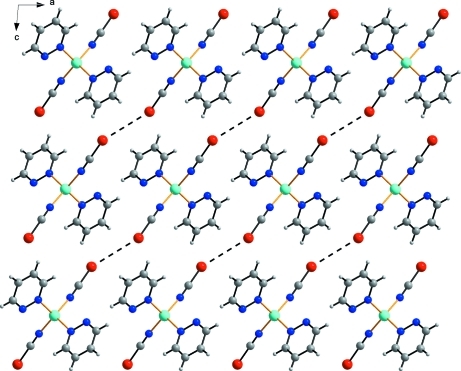
: Packing diagram of title compound with view along the crystallographic b axis. Intermolecular Se···Se interactions are shown as dashed lines.

## supplementary crystallographic information

### Comment

The structure determination of the title compound was performed as a part of a project on the synthesis of new selenocyanato coordination compounds (Wriedt & Näther, 2010). In our ongoing investigations we have reacted zinc(II)nitrate with potassium(I)selenocyanate and pyridazine in acetonitrile, which leads to the phase pure formation of bis(selenocyanato-*N*)-bis(pyridazine-*N*)zinc(II).

The title compound is isotypic to its thiocyanato analogon reported recently (Bhosekar *et al.*,  2010). In the crystal structure the zinc atoms are surrounded by two N-atoms of two symmetry equivalent pyridazine ligands and two N-bonded symmetry equivalent thiocyanato anions in a slightly distorted tetrahedral geometry (Fig. 1 and Tab. 1). The discrete complexes are arranged in layers parallel along the *ac* plane with Zn^II^···Zn^II^ distances of 8.0906 (6) Å along the *a* axis and of 9.0490 (7) or 9.3604 (7) Å along the *c* axis. Within these layers these complexes are further connected *via* weak Se···Se interactions of 3.8235 (9) Å (Fig. 2). Crystal structures of related thio- and selenocyanato compounds with pyridine as neutral coligand have already been described in literature (Zhu *et al.*, 2008; Boeckmann *et al.*, 2011).

### Experimental

The title compound was prepared by the reaction of 74.35 mg Zn(NO_3_)_2_ × 6 H_2_O (0.25 mmol), 72.0 mg KSeCN (0.50 mmol) and 18.1 µ*L* pyridazine (0.25 mmol) in 1.00 ml acetonitrile at RT in a closed 3 ml snap cap vial. After one week colourless blocks of the title compound were obtained.

### Refinement

All H atoms were located in difference map but were positioned with idealized geometry and were refined using a riding model with *U*_eq_(H) = 1.2 *U*_eq_(C) and with C—H = 0.93 Å.

### Crystal data

**Table:**

[Zn(NCSe)_2_(C_4_H_4_N_2_)_2_]	*F*(000) = 832
*M**_r_* = 435.51	*D*_x_ = 1.930 Mg m^−^^3^
Monoclinic, *C*2/*c*	Mo *K*α radiation, λ = 0.71073 Å
Hall symbol: -C 2yc	Cell parameters from 9054 reflections
*a* = 15.1521 (10) Å	θ = 2.3–27.0°
*b* = 5.6783 (4) Å	µ = 6.49 mm^−^^1^
*c* = 17.4855 (13) Å	*T* = 293 K
β = 94.981 (6)°	Block, colourless
*V* = 1498.74 (18) Å^3^	0.09 × 0.06 × 0.04 mm
*Z* = 4	

### Data collection

**Table:**

Stoe IPDS-2 diffractometer	1634 independent reflections
Radiation source: fine-focus sealed tube	1287 reflections with *I* > 2σ(*I*)
graphite	*R*_int_ = 0.028
ω scans	θ_max_ = 27.0°, θ_min_ = 2.3°
Absorption correction: numerical (*X-SHAPE* and *X-RED32*; Stoe & Cie, 2008)	*h* = −19→19
*T*_min_ = 0.373, *T*_max_ = 0.664	*k* = −7→7
9054 measured reflections	*l* = −22→22

### Refinement

**Table:**

Refinement on *F*^2^	Primary atom site location: structure-invariant direct methods
Least-squares matrix: full	Secondary atom site location: difference Fourier map
*R*[*F*^2^ > 2σ(*F*^2^)] = 0.046	Hydrogen site location: inferred from neighbouring sites
*wR*(*F*^2^) = 0.097	H-atom parameters constrained
*S* = 1.13	*w* = 1/[σ^2^(*F*_o_^2^) + (0.0314*P*)^2^ + 3.5658*P*] where *P* = (*F*_o_^2^ + 2*F*_c_^2^)/3
1634 reflections	(Δ/σ)_max_ < 0.001
87 parameters	Δρ_max_ = 0.49 e Å^−^^3^
0 restraints	Δρ_min_ = −0.39 e Å^−^^3^

### Special details

**Table:**

Geometry. All e.s.d.'s (except the e.s.d. in the dihedral angle between two l.s. planes) are estimated using the full covariance matrix. The cell e.s.d.'s are taken into account individually in the estimation of e.s.d.'s in distances, angles and torsion angles; correlations between e.s.d.'s in cell parameters are only used when they are defined by crystal symmetry. An approximate (isotropic) treatment of cell e.s.d.'s is used for estimating e.s.d.'s involving l.s. planes.
Refinement. Refinement of *F*^2^ against ALL reflections. The weighted *R*-factor *wR* and goodness of fit *S* are based on *F*^2^, conventional *R*-factors *R* are based on *F*, with *F* set to zero for negative *F*^2^. The threshold expression of *F*^2^ > σ(*F*^2^) is used only for calculating *R*-factors(gt) *etc*. and is not relevant to the choice of reflections for refinement. *R*-factors based on *F*^2^ are statistically about twice as large as those based on *F*, and *R*- factors based on ALL data will be even larger.

### Fractional atomic coordinates and isotropic or equivalent isotropic displacement parameters (Å^2^)

**Table:**

	*x*	*y*	*z*	*U*_iso_*/*U*_eq_	
Zn1	0.5000	0.29445 (12)	0.2500	0.0599 (2)	
N1	0.4362 (3)	0.4703 (8)	0.3215 (3)	0.0882 (13)	
C1	0.4026 (4)	0.5649 (9)	0.3694 (3)	0.0746 (13)	
Se1	0.35126 (4)	0.71204 (11)	0.44311 (3)	0.0895 (2)	
N11	0.5806 (2)	0.0691 (6)	0.31236 (18)	0.0529 (7)	
N12	0.6478 (3)	−0.0093 (8)	0.2760 (2)	0.0805 (12)	
C11	0.6957 (4)	−0.1819 (13)	0.3105 (5)	0.110 (2)	
H11	0.7432	−0.2417	0.2863	0.131*	
C12	0.6781 (5)	−0.2768 (11)	0.3808 (5)	0.108 (2)	
H12	0.7132	−0.3968	0.4033	0.129*	
C13	0.6107 (5)	−0.1931 (11)	0.4148 (4)	0.1007 (19)	
H13	0.5958	−0.2522	0.4616	0.121*	
C14	0.5640 (3)	−0.0171 (9)	0.3784 (3)	0.0747 (13)	
H14	0.5169	0.0465	0.4022	0.090*	

### Atomic displacement parameters (Å^2^)

**Table:**

	*U*^11^	*U*^22^	*U*^33^	*U*^12^	*U*^13^	*U*^23^
Zn1	0.0636 (4)	0.0529 (4)	0.0629 (4)	0.000	0.0043 (3)	0.000
N1	0.092 (3)	0.080 (3)	0.092 (3)	0.027 (2)	0.000 (2)	−0.027 (2)
C1	0.081 (3)	0.061 (3)	0.079 (3)	0.018 (2)	−0.009 (2)	−0.005 (2)
Se1	0.1106 (5)	0.0806 (4)	0.0785 (4)	0.0227 (3)	0.0144 (3)	−0.0112 (3)
N11	0.0485 (17)	0.0543 (19)	0.0559 (18)	0.0004 (14)	0.0047 (14)	−0.0038 (15)
N12	0.059 (2)	0.093 (3)	0.093 (3)	0.011 (2)	0.023 (2)	−0.005 (2)
C11	0.062 (3)	0.110 (5)	0.158 (7)	0.019 (3)	0.016 (4)	−0.023 (5)
C12	0.093 (4)	0.082 (4)	0.140 (6)	0.013 (4)	−0.039 (4)	0.011 (4)
C13	0.125 (5)	0.085 (4)	0.090 (4)	0.016 (4)	−0.005 (4)	0.019 (3)
C14	0.086 (3)	0.073 (3)	0.067 (3)	0.010 (3)	0.017 (2)	0.007 (2)

### Geometric parameters (Å, °)

**Table:**

Zn1—N1	1.925 (4)	N12—C11	1.332 (8)
Zn1—N1^i^	1.925 (4)	C11—C12	1.389 (11)
Zn1—N11^i^	2.022 (3)	C11—H11	0.9300
Zn1—N11	2.022 (3)	C12—C13	1.315 (9)
N1—C1	1.150 (6)	C12—H12	0.9300
C1—Se1	1.772 (5)	C13—C14	1.351 (8)
N11—C14	1.299 (5)	C13—H13	0.9300
N11—N12	1.324 (5)	C14—H14	0.9300
			
N1—Zn1—N1^i^	117.5 (3)	N12—C11—C12	123.2 (6)
N1—Zn1—N11^i^	111.40 (17)	N12—C11—H11	118.4
N1^i^—Zn1—N11^i^	106.96 (16)	C12—C11—H11	118.4
N1—Zn1—N11	106.96 (16)	C13—C12—C11	118.4 (6)
N1^i^—Zn1—N11	111.40 (17)	C13—C12—H12	120.8
N11^i^—Zn1—N11	101.48 (18)	C11—C12—H12	120.8
C1—N1—Zn1	173.8 (4)	C12—C13—C14	116.8 (6)
N1—C1—Se1	179.7 (6)	C12—C13—H13	121.6
C14—N11—N12	121.1 (4)	C14—C13—H13	121.6
C14—N11—Zn1	124.3 (3)	N11—C14—C13	124.3 (5)
N12—N11—Zn1	114.1 (3)	N11—C14—H14	117.8
N11—N12—C11	116.2 (5)	C13—C14—H14	117.8

Symmetry codes: (i) −*x*+1, *y*, −*z*+1/2.
